# ProteoGyver: a fast, user-friendly tool for routine QC and analysis of MS-based proteomics data

**DOI:** 10.1093/bioinformatics/btag050

**Published:** 2026-01-30

**Authors:** Kari Salokas, Salla Keskitalo, Markku Varjosalo

**Affiliations:** Institute of Biotechnology, HiLIFE Helsinki Institute of Life Science, University of Helsinki, Viikinkaari 9, 00790 Helsinki, Finland; Institute of Biotechnology, HiLIFE Helsinki Institute of Life Science, University of Helsinki, Viikinkaari 9, 00790 Helsinki, Finland; Institute of Biotechnology, HiLIFE Helsinki Institute of Life Science, University of Helsinki, Viikinkaari 9, 00790 Helsinki, Finland

## Abstract

Mass spectrometry-based proteomics generates increasingly large datasets requiring rapid quality control (QC) and preliminary analysis. Current software solutions often require specialized knowledge, limiting their routine use. We developed ProteoGyver (PG), an accessible, lightweight software solution designed for rapid QC and preliminary proteomics data analysis. PG provides automated QC metrics, intuitive graphical reports, and streamlined workflows for whole-proteome and interactomics datasets, significantly lowering the barrier to regular QC practices. The platform includes additional tools such as *MS Inspector* for longitudinal chromatogram inspection and *Colocalizer* for microscopy data. PG is easily deployed as a Docker container or standalone Python installation. PG is open-source and freely available in dockerhub and source code in github at github.com/varjolab/Proteogyver.

**Availability and implementation:**

PG image and source code are available in github and dockerhub under LGPL-2.1.

## 1 Introduction

Mass spectrometry-based proteomics, the large-scale study of protein function, location, interactions, modifications, and perturbations under different conditions, is a cornerstone of modern biological and biomedical research. Advances in liquid chromatography (LC) and mass spectrometry (MS) instrumentation enables researchers to identify over 10 000 proteins and hundred thousand peptides with high throughput. However, this increased data throughput presents challenges in data management as well as in performing rapid quality control (QC) and analysis.

QC is essential to ensure that proteomics MS data meet the required standards for drawing robust conclusions in downstream analysis. The need for more robust QC is well recognized and has been raised as an ethical issue affecting both the field and the reproducibility of results produced (Mann *et al.* 2021, Rozanova *et al.* 2023). For example, recent studies in human cerebrospinal fluid (CSF) proteomics have demonstrated that inadequate QC can lead to biomarker misidentification, directly impacting the reliability of clinical diagnostics and therapeutic research outcomes (Schilde *et al.* 2018, Rozanova *et al.* 2023).

While instrument operators are well attuned to the performance of their instruments and have a long history of ensuring good technical quality in data, QC relating to sample material, collection, and handling is often overlooked, or inconsistently applied. Similarly, preliminary analysis steps for proteomics samples often follow a predictable routine, e.g. clustering, PCA, and differential abundance analysis are typically performed to gain an overview of the identified proteome and sample groups.

For protein-interactomics datasets, where the focus is on protein-protein interactions (PPIs), additional steps are required. Identification of high-confidence interactions from data predominantly composed of background and contaminants is the foremost challenge of interactomics. Evaluating the efficacy of filtering approaches can be helped by e.g. mapping known interactions, protein complexes, Gene Ontology (GO) annotations, and analyzing protein identification profiles across the sample set. In addition to QC, the rapid and routine employment of these steps aids the identification of not only failed samples, but also critical follow-up experiments to be performed. Despite the routine nature of these steps, they are often carried out with fragmented, laborious, or non-standardized approaches, which limit reproducibility and reliability in proteomics studies.

With these considerations in mind, we set out to develop a quick and easy way to calculate and visualize QC metrics after protein identification. We identified a need for a low-threshold and highly available web-based platform, which offers these QC metrics and preliminary analysis steps as one package, and identically for all users. The resulting open-source ProteoGyver platform (PG) is available as a Docker container. It features a web-based user interface with highly automated QC and analysis workflows, making these processes accessible even to non-specialists.

By simplifying early-stage analysis, PG improves error detection, enables earlier follow-up planning, and frees researchers to focus on deeper biological questions.

## 2 ProteoGyver

**Figure 1:**
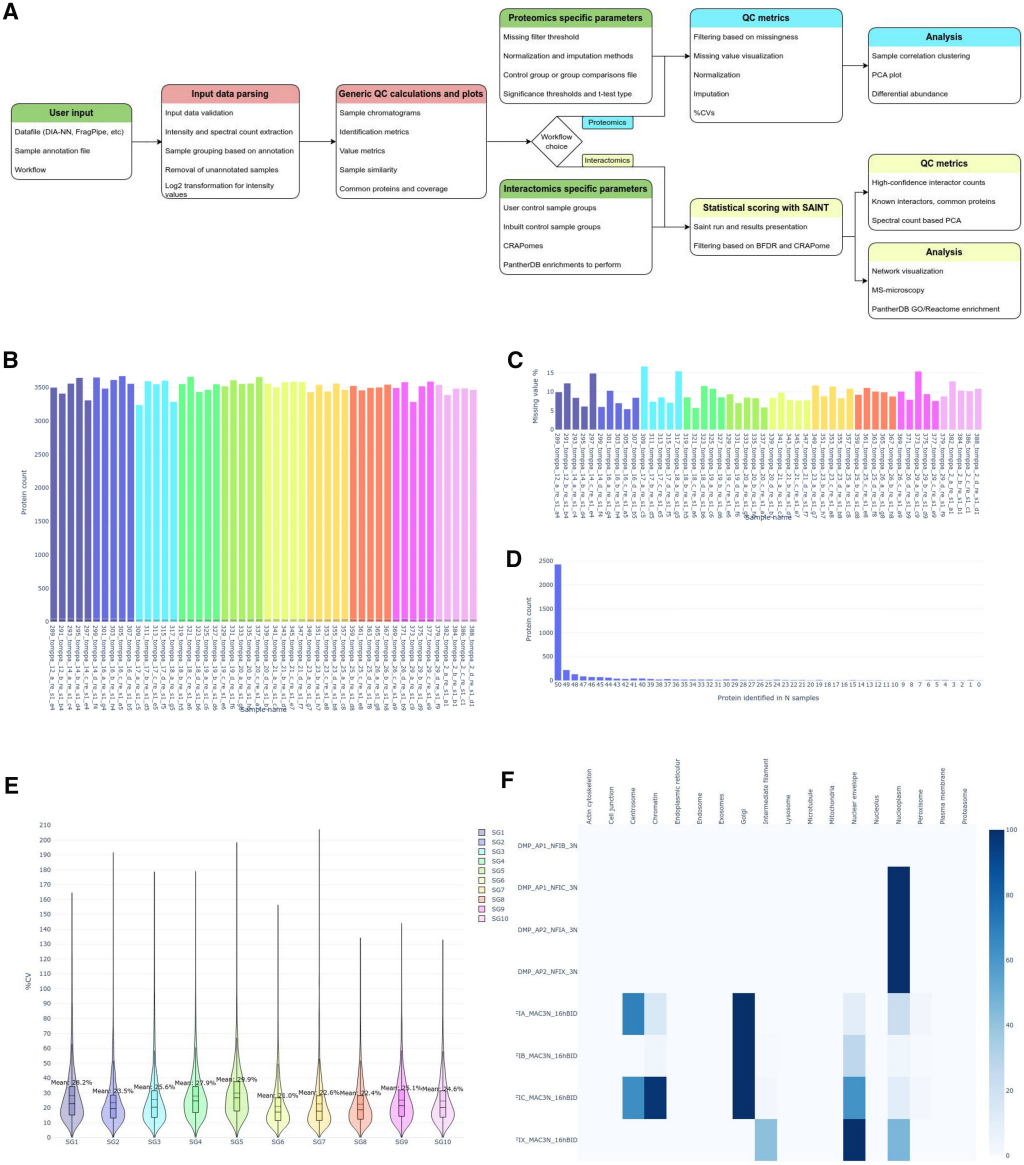
The ProteoGyver data-processing workflow with representative generic and workflow-specific quality-control metrics. (A) Flowchart illustrating the data-processing steps implemented in ProteoGyver. (B) Protein identification counts per sample. (C) Percentage of missing values per sample. (D) Plot showing the number of samples in which each protein was identified. (E) Coefficient of variation (CV) plot for each sample group. (F) MS-microscopy heatmap depicting the predicted subcellular localization of each bait protein. Panels B–D represent generic QC metrics generated for all datasets, whereas panel E is specific to the proteomics workflow and panel F is specific to the interactomics workflow.

PG was developed in Python and R, and its source code is available at github.com/varjolab/Proteogyver. The platform is easily deployable as a Docker container, which is available via dockerhub at hub.docker.com/r/ksal/proteogyver. The update companion container is available from dockerhub as well: hub.docker.com/r/ksal/pg_updater. Figure 1 illustrates the workflows and features currently available on the platform. Usage documentation and examples utilizing data from previously published papers (Hulmi *et al.* 2025, Malaymar Pinar *et al.* 2025) are available on github, and ReadTheDocs (proteogyver.readthedocs.io/).

### 2.1 MS data pre-analysis

In order for PG to display chromatogram data in QC analysis and MS inspector, MS runs need to be pre-analyzed with the included MSParser script package. The script extracts chromatogram info, as well as sample metadata, from raw MS files [currently ThermoFisher .raw and Bruker (Willems *et al.* 2021) .d formats are supported] and saves it in json format, which PG can automatically ingest. The MS data pre-analysis is an optional step: without MS data, PG will still work, but all chromatogram -related functions (MS inspector, TIC graphs) is nonfunctional.

### 2.2 Data submission

PG requires two primary inputs: (i) A data file, consisting of proteomics results, e.g. a generic intensity or spectral count matrix, or a report file from FragPipe or DIA-NN. (ii) A sample table, which lists which sample (e.g. a column name from the data matrix) belongs to which sample group. For interactomics, a bait uniprot column is optional, but highly encouraged (without it, known interactions cannot be mapped). Optionally (on by default), common contaminant proteins (Frankenfield *et al.* 2022) will be removed from the data before any processing is done. Additional proteomics or interactomics-specific input options will be presented after initial data files are uploaded and QC metrics and plots processed. A specific emphasis was placed on minimizing the amount of information the user needs to submit. Therefore, PG aims to identify automatically the format uploaded, and whether data includes intensities, spectral counts, or both. For the same reason, PG is intended to be the first step in the analysis of a dataset, a tool to identify issues early and perform preliminary analysis.

### 2.3 Pipeline module for automated processing

For automated processing, PG includes a pipeline mode. Given that it is intended to be used as part of an automated processing pipeline, e.g. directly after a FragPipe analysis is finished on a server, it takes as input a minimum of three files: pipeline definition file, data file, and sample table. The latter two are identical to the GUI inputs, while the pipeline definition file specifies, which workflow should be run, with what data, and which options for e.g. imputation in proteomics, or control samples and enrichments in interactomics. The pipeline module is set to monitor a specified input directory, and results, or errors, will be generated in the same directory, together with a pipeline.success or pipeline.failure -file to indicate the end of processing. Pipeline mode is also available through an API interface, which takes in the same three files as before, and can be polled to identify when processing is done, after which results may be obtained with another API call.

### 2.4 Automated quality control

PG performs automated QC analysis on all submitted data. From the data, PG will present sample run chromatograms (if available in the database) as a time series for inspection (to detect e.g. drift issues), and generate graphs for common QC metrics, such as identification counts, commonly identified protein groups, shared identifications, identification coverage, missing values, and intensity/spectral count metrics. All graphs are accompanied by short figure legends explaining the purpose of the graph and how to interpret the success or failure of the sample set based on it. After the generic QC workflow has finished, dataset-specific workflows become available. QC graphs will remain visible throughout, to ensure the user has a chance to inspect the graphs before seeing the more specific analysis results.

### 2.5 Proteomics workflow

The Proteomics workflow covers data handling and analysis steps that are nearly always performed for datasets where the interest is in the whole proteome complement of the sample material. The data is assumed to consist of intensity values. As data from various search programs are often already normalized [e.g. by using the MaxLFQ algorithm (Cox *et al.* 2014)], PG does not normalize the data by default.

The workflow begins with filtering out proteins, which have not been identified consistently (by default, proteins appear in at least two thirds of the samples of at least one sample group). Next, data are optionally normalized [via either quantile (van der Sande and van Heeringen 2025), vsn (Zhang *et al.* 2018), or median] and missing values are imputed [by default QRILC (Lazar *et al*. 2022), other methods are also available, including random forest]. All methods are detailed in the full documentation available at ReadTheDocs and github. At this point, data preprocessing is considered complete, and PG will then generate PCA plots, perform clustering analysis, and visualize differential abundance (using user-supplied control groups or a comparison file) via volcano plots and heatmaps. The volcano plots are generated with cutoffs based on log2 fold change of intensity values (by default, −1 and 1), and adjusted (Benjamini-Hochberg) *P*-value (by default, 0.01 threshold).

### 2.6 Interactomics workflow

For interactomics, PG offers built-in GFP control sets and identifies control samples from user input. Built-in control sets can serve as “CRAPomes” for fold-change-based filtering (Mellacheruvu *et al.* 2013, Salokas *et al.* 2022). Users can optionally choose to perform enrichments via PantherDB (Mi *et al.* 2019, Thomas *et al.* 2022), after obtaining high-confidence interactors. The workflow runs SAINTexpress (Teo *et al.* 2014), then applies user-selected filters (Bayesian FDR and CRAPome filtering parameters). After the final set of high-confidence interactors have been filtered out, the user is presented with more QC plots detailing commonly copurified proteins or contaminants, as well as mapped known interactors (Oughtred *et al.* 2021, del Toro *et al.* 2022), cytoscape-based (Shannon *et al.* 2003) network plot, MS-microscopy (Liu *et al.* 2018), and enrichments via PANTHER API (Thomas *et al.* 2022).

### 2.7 Downloadable output

For the QC and preliminary analysis workflows, downloadable data includes data summaries, all graphs generated (as PNG, PDF, and interactive HTML) and the data used for the graphs, together with intermediate data from steps such as imputation or filtering. The output also includes data and metrics useful for debugging in JSON format, as well as an output guide document in HTML-based output guide, and a document specifying all options selected during the analysis. Software and data versions are also specified.

### 2.8 Reproducibility

To ensure reproducibility, database snapshots can be exported to directories specified in the configuration file periodically. Database updates are also not automatic by default, but performed via a secondary docker container (pg_updater), which can be scheduled to run at intervals. All operations relating to randomness (e.g. in imputation) use a fixed random seed, and downloadable output specifies each option chosen during a given workflow.

### 2.9 MS inspector

Over the course of proteomics experiments, it is vital to ensure that the data quality of the raw data is comparable and consistent. Depending on experiment type, this can be a challenge: mostly instrument operators handle identification of issues and ensuring each run is technically good. However, different sample types and materials behave differently over the course of an experiment, and some more subtle changes are only visible when observed as a time series of all the runs of a dataset. To make the inspection of key MS raw data attributes straightforward and fast, we decided to bundle a tool for the job with PG: the MS Inspector.

This tool leverages precalculated metrics and extracted chromatograms from the PG database to present them in a uniform way as a time series (Fig. 1A, available as supplementary data at *Bioinformatics* online). MS Inspector lets the user choose any set of runs based on either various parameters or as a list of run IDs or file names. It then presents an animated view of chromatograms, as well as several supplementary plots: area under the curve for the chromatogram, and plots for mean and maximum intensities. While MS runs require pre-analysis with the included MSParser tool, examples of parsed runs from previous publications (Hulmi *et al.* 2025, Malaymar Pinar *et al.* 2025) are included in the database by default. These metrics and their change over time as the run series progresses are critical to verify the quality of the entire dataset. Currently only time and *m*/*z* data are used for plotting and analysis, however incorporating ion mobility would be an interesting future prospect as well.

### 2.10 Colocalizer

The Colocalizer tool is designed to analyze confocal microscopy data, specifically by taking in .LIF files. It generates a colocalization map of different fluorescent markers based on the user’s choice of z-slice and channels (Fig. 1B, available as supplementary data at *Bioinformatics* online). The tool allows the user to choose how to create the colocalization map (Mäntylä *et al.* 2016) by either multiplying or adding channels together, which provides flexibility depending on the user’s experimental needs. Additionally, Colocalizer offers the option of using either linear or logarithmic scaling for the final image, allowing for better visualization of differences in fluorescence intensity. This tool is essential for visualizing the spatial overlap between different markers, enabling researchers to assess the degree of colocalization, which can provide important insights into biological interactions at the cellular level.

### 2.11 Automated update companion

A second container for database updates is also included. It performs automated updates of the database, including the incorporation of new MS runs and periodical updates of UniProt, IntAct (del Toro *et al.* 2022), and BioGRID (Oughtred *et al.* 2021) databases. This ensures the data stays up to date and relevant, while still requiring a decision about how and when to schedule the updates, ensuring no unexpected database updates occur. The updater container is also available in dockerhub: hub.docker.com/r/ksal/pg_updater, and source code in the proteogyver github repository.

## 3 Conclusions

ProteoGyver consists of three sets of tools: QC/Preliminary data analysis, MS Inspector, and Colocalizer, developed largely to aid in common tasks encountered during proteomics experiments. Source code is available on github and zenodo, and the container on dockerhub. For more expert users, PG offers a framework to relatively easily add functionality in the form of additional analysis steps, or additional pages for different types of analysis, including embedding pre-existing tools. The combination of tools such as MS Inspector and Colocalizer within PG demonstrates its versatility in tackling different types of data, and the ease with which different, existing or newly created tools can be added. The goal of PG is to be a comprehensive, daily-use platform that streamlines the process of QC and analysis.

Future prospects for PG include the addition of a generalized PTM workflow for PTM level QC analysis and preliminary data analysis, as well as several optimizations for back-end data handling and computation. In addition, version 1.6 is planned to include automated generation of a unified pdf report from same data that is already exported.

In comparison to other, more specialized tools for deeper analysis of either proteomics results, or raw data from the mass spectrometer, PG aims to sit in the middle: to provide easy to understand QC metrics on the dataset level. This comes with many limitations: while chromatograms can point out when something went wrong, PG cannot provide answers as to what and why. The same applies to results analysis: PG can identify that a subset of samples or the whole dataset has low identifications or a high variability, but it cannot tell the user whether it’s due to e.g. wrong parameters in MSFragger, or problems with the FASTA library.

At the same time, PG does provide rudimentary analysis for both proteomics (such as differential abundance and clustering) and interactomics [e.g. functional enrichment via PANTHER (Thomas *et al.* 2022), MS microscopy (Liu *et al.* 2018)], but it cannot identify for the user, whether the analysis is adequate for their use case, or whether an approach e.g. without imputation would be more robust. The analysis in PG is well suited for routine samples and simple interactomics datasets, and is mainly designed to point out problems as early as possible, and provide a generalized overview with which to judge data quality and continue into downstream analysis.

In conclusion, PG addresses longstanding QC and analysis challenges by providing streamlined, user-friendly workflows accessible even to non-specialists. Current solutions for QC and preliminary analysis often rely either on fragmented tools or workflows that require expertise to use effectively. In comparison to other available tools such as MSstats (Kohler *et al.* 2023) and FragPipe analyst (Hsiao *et al.* 2024), PG aims to deliver workflows for routine and rapid QC and preliminary data analysis with minimal threshold for daily use, and with as wide a range of options for hosting the platform as possible. This reduces the need for specific technical knowledge, making it more accessible to non-specialists. Particularly if set up on a server, PG offers an easy to use, low-threshold workflow for quality assurance and preliminary analysis that is immediately available for use for a wide range of users.

## Supplementary Material

btag050_Supplementary_Data

## Data Availability

The data underlying this article are available in zenodo at https://doi.org/10.5281/zenodo.15745814.
