# Supplementary figures and images for: ProteoGyver: a fast, user-friendly tool for routine QC and analysis of MS-based proteomics data
Source: Bioinformatics. 2026 Jan 30;42(2):btag050. doi: 10.1093/bioinformatics/btag050 (PMC12910381; doi:10.1093/bioinformatics/btag050)

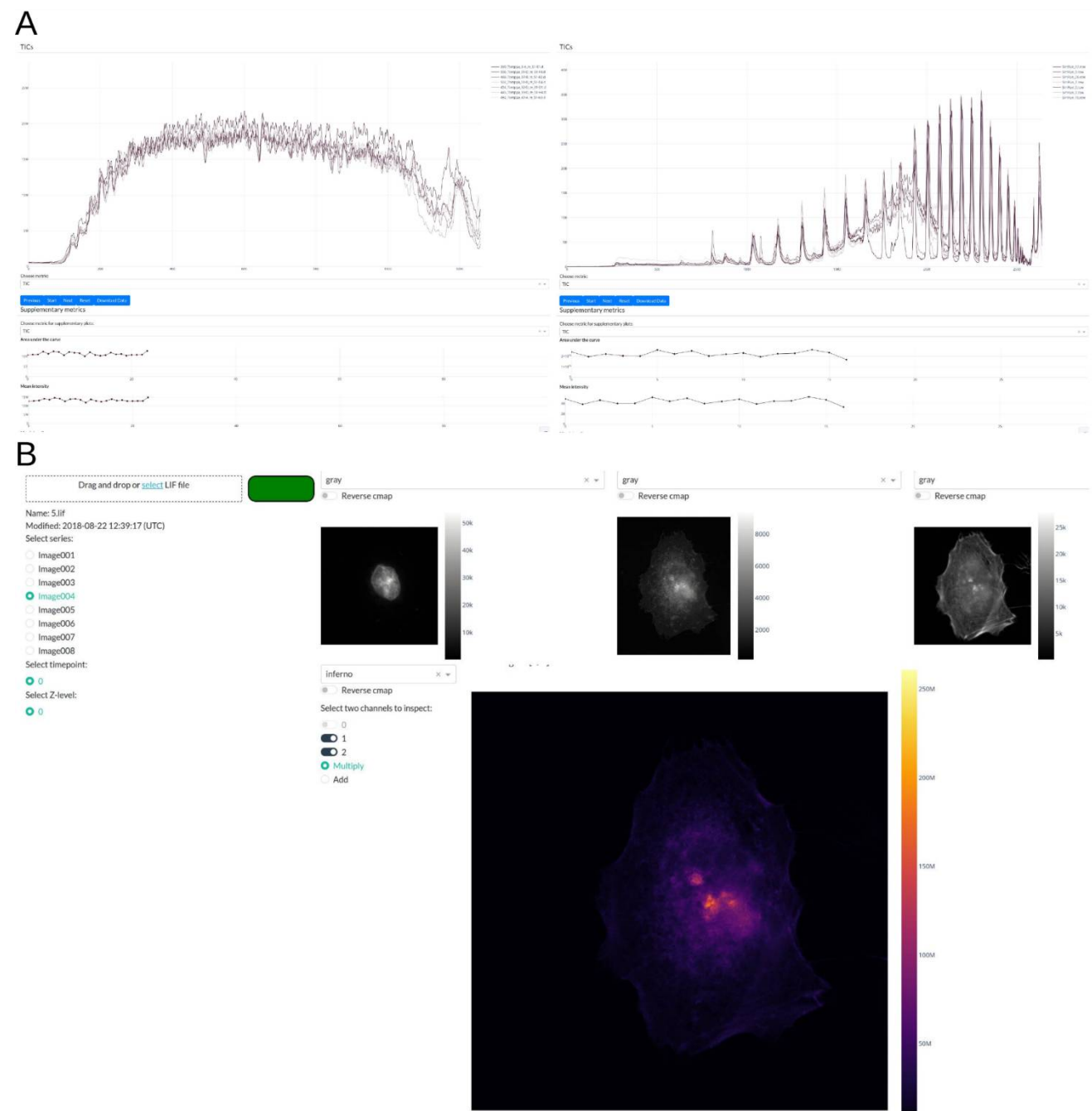

Supplement: btag050_Supplementary_Data [file btag050_supplementary_data.pdf]
